# Advanced Glycation End Products Play Adverse Proinflammatory Activities in Osteoporosis

**DOI:** 10.1155/2014/975872

**Published:** 2014-03-20

**Authors:** Roberta Sanguineti, Alessandra Puddu, François Mach, Fabrizio Montecucco, Giorgio Luciano Viviani

**Affiliations:** ^1^Department of Internal Medicine, University of Genoa, 6 Viale Benedetto XV, 16132 Genoa, Italy; ^2^Division of Cardiology, Foundation for Medical Researches, Faculty of Medicine, Geneva University Hospitals, 64 Avenue de la Roseraie, 1211 Geneva, Switzerland; ^3^First Clinic of Internal Medicine, Department of Internal Medicine, University of Genoa School of Medicine, IRCCS Azienda Ospedaliera Universitaria San Martino-IST Istituto Nazionale per la Ricerca sul Cancro, 6 Viale Benedetto XV, 16132 Genoa, Italy; ^4^Division of Laboratory Medicine, Department of Genetics and Laboratory Medicine, Geneva University Hospitals, 4 Gabrielle-Perret-Gentil, 1205 Geneva, Switzerland

## Abstract

Osteoporosis is a major public health burden that is expected to further increase as the global population ages. In the last twenty years, advanced glycation end products (AGEs) have been shown to be critical mediators both in the pathogenesis and development of osteoporosis and other chronic degenerative diseases related to aging. The accumulation of AGEs within the bone induces the formation of covalent cross-links with collagen and other bone proteins which affects the mechanical properties of tissue and disturbs bone remodelling and deterioration, underlying osteoporosis. On the other hand, the gradual deterioration of the immune system during aging (defined as immunosenescence) is also characterized by the generation of a high level of oxidants and AGEs. The synthesis and accumulation of AGEs (both localized within the bone or in the systemic circulation) might trigger a vicious circle (in which inflammation and aging merged in the word “Inflammaging”) which can establish and sustain the development of osteoporosis. This narrative review will update the molecular mechanisms/pathways by which AGEs induce the functional and structural bone impairment typical of osteoporosis.

## 1. Introduction

Bone diseases represent a major socioeconomic issue as recently recognized by The World Health Organization [1]. The development of innovative bone-healing strategies has been described as a prerequisite for the successful treatments against bone defects [2, 3]. Among the wide spectrum of bone disorders, osteoporosis has emerged as a medical and socioeconomic threat [2]. Although it is accepted that more than 8.9 million fractures annually worldwide are caused by osteoporosis, they are often diagnosed only after the first clinical fracture has occurred because bone loss arises insidiously and is initially asymptomatic. The lifetime fracture risk of a patient with osteoporosis has been estimated to be in the order of 30–40%, which is very close to the risk for coronary heart disease [4]. Moreover, in addition to pathologic fractures, osteoporosis carries a considerable risk of disability due to serious medical complications. With the aging of the population, the prevalence of osteoporosis is expected to further increase [3].

Osteoporosis is characterised by a systemic impairment of bone mass, strength, and microarchitecture, which increases the propensity of fragility fractures. This pathological condition, whose aetiology is attributed to various endocrine, metabolic, and mechanical factors, can occur at any age of life, but it is predominantly found in elderly and diabetic patients [3, 5]. As a skeletal disorder, osteoporosis results from an heterogeneous group of abnormal processes leading to low bone mass and bone microarchitectural disruption [4]. Low bone mass may result from increased bone resorption and/or reduced bone formation during remodelling, being commonly accepted that the first has a higher impact on osteoporosis development. Despite that the osteoporotic bone is normally mineralized, there is a disruption of its normal trabecular bone loss and microarchitecture and an increased cortical porosity [4]. Recently, growing understanding of bone remodelling process suggests that factors involved in inflammation are linked with those critical for bone physiology and remodelling, supporting the hypothesis that inflammation significantly contributes to the pathogenesis of osteoporosis [6–12].

## 2. Pathophysiology of Osteoporosis

Bone is a permanently regenerating organ, which is continually renewed in a complex process of formation and resorption [13]. Bone remodeling is a physiological process necessary to maintain the quality and strength of the skeleton by removing old bone and replacing it with a young matrix [13, 14]. It occurs in the microscopic basic multicellullar units (BMU), mainly composed of osteoblasts, osteoclasts, and osteocytes. The normal bone remodelling couples bone resorption and bone formation, which are primarily mediated by osteoclasts and osteoblasts, respectively [15, 16].

Osteocytes, star-shaped cells derived from osteoblasts trapped in their secreted matrix, play an essential role in coordinating bone remodeling detecting microcracks, mechanical strain, and the changes in the bone hormonal milieu, communicating these alterations to the bone-lining cells, which in turn initiate bone resorption and formation [17, 18]. Osteoclasts are multinucleated cells derived from monocyte/macrophage lineage and are the only type of cells capable of resorbing bone. The rate of bone resorption is determined by the number and activity of osteoclasts. During bone resorption, osteoclasts adhere to the bone matrix forming a deeply folded membrane and secrete protons and hydrolytic enzymes in a small cavity called Howship's lacunae, formed from the digestion of the underlying bone [19]. The lacuna is then demineralized by the acidic environment due to proton secretion, leading to the exposure of bone organic components, such as collagen, to the hydrolytic enzymes, resulting in degradation of the organic components.

Osteoblasts and their constituent progenitor cells migrate to the newly resorbed surface where they produce an osteoid matrix and mineralize the osteoclast-orchestrated cavities. The formation phase is followed by the osteoblasts which lay down bones until the resorbed bone is completely replaced with unmineralized bone matrix [20]. Primary and secondary mineralization completes this remodelling process. At this stage, the majority of osteoblasts die by apoptosis or become embedded in bone matrix as osteocytes [13, 14, 17, 18, 21].

## 3. Advanced Glycation End Products (AGEs) and Osteoporosis 

In the past decade, studies investigating the pathogenesis of osteoporosis allowed the identification of several tissue, cellular, and molecular processes. Recent evidence supports the hypothesis that protein glycation may affect bone remodelling [22, 23]. The nonenzymatic glycation reactions cause generation and accumulation of AGEs, which, in turn, induce tissue damage through the structural modification of proteins, the stimulation of cellular responses via specific receptors for AGEs, and the generation of reactive oxygen intermediates [22].These deleterious processes are attributed to their chemical, pro-oxidant, and inflammatory actions that may contribute to increase oxidative stress and as a final result impair organ function [24–27]. AGEs are formed during ageing as a physiological and inevitable process* in vivo*, but their excessive generation and accumulation are found in osteoporosis [28].

The content of AGEs increases during aging in all tissues including bone and contributes to the structural and functional changes of bone proteins through a process called cross-linking, which occurs mainly on long-lived matrix proteins (such as collagen I), leading to intra- or intermolecular cross-links, partially explaining the deleterious effects of AGEs on bone biomechanical properties [29–33]. Bone slow turnover process favours the establishment of these tissue alterations since matrix proteins are exposed to the extracellular environment for extended times, leading to modifications by nonenzymatic glycation [7, 23, 28, 32, 34].

The modifications of bone proteins may result in functional alterations of osteoclasts and osteoblasts, and these changes might be of significant pathophysiological importance in the development of disease [35–37].

General mechanisms through which AGEs contribute to induce damage include (1) formation of cross-links with targeted proteins contained in the tissues where they accumulate, permanently altering cellular structure and inducing formation and accumulation of irreversibly cross-linked heterogeneous protein aggregates; (2) interaction with several specific receptors increasing oxidative stress and inflammation [38]. Among those molecules, the receptor for advanced glycation end products (RAGE) is the best characterized receptor which initiates the intracellular signalling that disrupts cellular function through its recognition and binding of AGEs [39].

Multiple RAGE isoforms seem to arise through alternative splicing and/or proteolysis [38, 40]. Three different isoforms have been described: a “full-length” transmembrane isoform (responsible for intracellular signal transduction), an isoform lacking the transmembrane and signalling domain (commonly referred to as soluble RAGE (sRAGE), i.e., thought to be produced by proteolytic cleavage from cell surface receptor by actions of disintegrin and metalloproteinase domain-containing proteins (ADAMs)) and endogenous secretory RAGE ((esRAGE), in which a natural gene alternative splicing occurs). These two forms of soluble RAGE are hypothesized to counteract the detrimental action of the full-length receptor contributing to the removal/neutralization of circulating ligands thus functioning as a decoy [38, 39].

Other receptors, such as AGE-R1 (oligosaccharyl transferase-48), -R2 (80K-H phosphoprotein), and -R3 (galectin-3), and the class A of macrophage scavenger receptor types I and II were shown to recognize and bind AGEs, but they were unable to transduce intracellular signals. Instead, they are involved in the clearance and possible detoxification of AGEs [41, 42]. RAGE is a multiligand member of the immunoglobulin superfamily widely expressed in a range of cell types and tissues at very low levels in physiological condition, while an increased expression was noted in disease states, such as diabetes, neurodegenerative disorders, and autoimmune/inflammatory conditions [41].

The RAGE signaling pathway can be initiated by a heterogeneous group of proinflammatory ligands including AGEs, amphoterin, S100/calgranulins, and *β*-amyloid peptide [39, 43, 44]. RAGE binding by AGEs did not accelerate their clearance and degradation but conversely induce sustained postreceptor signalling, including activation of the nuclear factor-kappa B (NF-kB) pathway, involved in the control of DNA transcription to induce cellular response to injury, and mitogen-activated protein (MAP) kinases which participate in signal transduction pathways that control intracellular events including acute responses to hormones and major developmental changes in organisms [44]. Furthermore, RAGE increases the production of reactive oxygen species, activates NADPH oxidase, increases expression of adhesion molecules, and upregulates inflammation with prolonged cellular dysfunction and localized tissue destruction [40, 44, 45].

The RAGE promoter contains NF-kB binding sites that are involved in the regulation of RAGE expression itself. Therefore, enhanced expression of NF-kB results in increased RAGE expression, thereby establishing a vicious circle by prolonging NF-kB activation [45].

RAGE expression occurs in an inducible manner and is upregulated at sites where its ligands accumulate [39]. For this reason, sustained RAGE cell expression in proximity to its ligands leads to chronic activation of inflammation and tissue damage. Inflammatory mediators that are upregulated through AGE- and NF-kB-mediated pathway include tumor necrosis factor-alpha (TNF-*α*), interleukin-1 (IL-1), IL-6, and C-reactive protein (CRP) [39]. Taken together, these considerations suggest that RAGE plays multiple roles in executing the signal transduction mechanisms initiated by ligands binding and that RAGE activation contributes to perpetuation of AGEs and proinflammatory ligands synthesis, both by creating a microenvironment conducive for ligands production (oxidative stress and inflammation) and by suppressing protective mechanisms. Bone, being a tissue containing long lifetime proteins such as type I collagen (COL I) and with a slow turnover, is exceptionally susceptible to develop and accumulate AGE modification over time [46, 47]. COL I is the most abundant type of collagen [48]. It is widely distributed in almost all connective tissues with the exception of hyaline cartilage [48]. In addition, COL I comprises approximately 95% of the entire collagen content of bone, where collagen fibrils are stiffened by integration of the mineral phase during the mineralization process, and represents about 80% of the total bone proteins [49]. COL I molecules are precisely aligned within the fiber in a quarter-staggered end-overlap fashion. This arrangement provides holes within the fiber for nucleation of the calcium apatite crystals, and these crystals then grow parallel to the collagen fibrils. The structure and organization of collagen fibrils limit the size of crystals and control their orientation that is fundamental in determining bone mechanical properties [50].

Spontaneous nonenzymatic glycation reactions lead to AGE formation and accumulation which are characterized by a resistance to proteolytic degradation and decreased solubility: the consequence of this accumulation is induction of structurally altered forms of collagen molecule with disrupting protein regulation or function [48, 51]. An increased concentration of AGEs with age in cortical and trabecular bone is negatively associated with bone density and mineralization. In addition, it induces bone cell functional impairment and affects cortical and trabecular biomechanical properties [31, 48, 51–53]. Cross-linking between collagen and AGEs alters the mechanical properties of bone, disturbing its remodeling and inducing a deterioration of tissue quality by increasing stiffness and fragility [32, 34, 54].

Katayama and colleagues showed that AGE-modified collagen was able to regulate osteoblast proliferation and differentiation inhibiting their phenotypic expression, suggesting that the glycation of bone proteins is able to affect osteoblast-mediated neoapposition of bone mineral matrix [28, 55–57].

AGEs were shown to affect osteoblast proliferation and differentiation via specific binding sites [23, 58]. Moreover, in these cells, AGE/RAGE binding was able to elicit the activation of NF-kB, resulting in an increased expression of cytokines, growth factors, and adhesion molecules [59] and contributing to the activation of inflammatory processes linked to bone remodeling disorder [32].* In vitro* studies from osteoblastic cell cultures have shown that AGE-modified collagen was able to inhibit the proliferation and differentiation [34, 36, 37, 60, 61]. Furthermore, Yamamoto and coworkers showed that the treatment with AGE-modified bovine serum albumin in cultured human osteoblast-like cells resulted in a significantly reduced synthesis of COL I and osteocalcin (a noncollagenous bone protein produced solely by osteoblasts and implicated in bone mineralization and calcium ion homeostasis) [62]. More recently, in human osteoblast primary cultures, we have also demonstratedthat pentosidine (a well-characterized AGE) exerts a dose-dependent detrimental activity on osteoblast function and inhibits bone nodule formation [58]. AGEs and their receptor RAGE might elicit oxidative stress generation and subsequently evoke inflammatory responses in osteoblasts and osteoclasts, thereby being involved in both vascular calcification and osteoporosis in diabetes [63]. The detrimental effect of AGEs on osteoblast function might also increase apoptosis [64] via various autocrine and paracrine pathways [65], involving IGF-I and its binding proteins [60], IL-6 and transforming growth factor-*β* (TGF-*β*) [61].

These considerations suggest that the real effects of AGEs on bone cells require further clarifications. A key opened question in the biology of AGEs is whether they are only innocent bystander biomarkers of diseases in which they accumulate, or whether AGEs actively contribute to the disease, thereby altering gene programs and cellular fate and function. The answer to this pivotal question is under active investigation and is eagerly warranted.

## 4. The Effects of Biphosphonates on AGEs

The antiresorptive agents, bisphosphonates (BP), have become the most commonly used family of antiosteoporotic drugs [66]. They are synthetic analogs of inorganic pyrophosphates that bind to the divalent calcium ion (Ca2+) in the hydroxyapaitite crystal of bone. Here, nitrogen-containing bisphosphonates are able to decrease osteoclast activity and survival by repressing farnesyl diphosphate synthase, an enzyme in the mevalonate pathway that is important for the synthesis of osteoclast cell regulatory proteins. Osteoclasts can no longer function without these proteins and bone resorption is substantially reduced. With decreased osteoclastic activity, resorption sites are reduced. Thus, the risk that an external mechanical load could impart damage, leading to trabecular instability and to catastrophic structural failure, would be reduced [66].

BP can be grouped into two classes with different molecular mechanisms of action. Nitrogen-containing bisphosphonates (i.e., alendronate, risedronate, pamidronate, and zoledronate) are the most potent kind and act by inhibiting the mevalonate pathway, thereby preventing prenylation of small GTPase signaling proteins. On the other hand, bisphosphonates that lack a nitrogen in their chemical structure (i.e., etidronate and clodronate) are less potent and have a different mode of action that may involve the formation of cytotoxic metabolites or inhibition of protein tyrosine phosphatases [66].

BP have been shown to affect bone metabolism mainly by inhibiting osteoclastic recruitment, activity, and survival. In order to reduce the amount of bone resorbed, BP prevent osteoclasts attachment to the bone surface, also inducing early apoptosis [67, 68]. Finally, BP have been also shown to positively influence osteoblastic development and bone-forming activity [69–72]. Although the precise mechanisms have not been elucidated yet, part of these actions might result from the reduction of RANKL expression by osteoblasts and/or bone marrow stromal cells, which in turn decreases osteoclastogenesis and bone resorption [73]. The suppression of resorption in bone leads to a reduction in remodeling space, an increased average tissue mineralization, and an altered tissue mineral density distribution [74], which, in turn, decreases fracture risk [75]. Therefore, BP treatment is associated with low bone turnover via the interruption of the tightly coupled bone-renewing synchrony of osteoclasts and osteoblasts. On the other hand, BP significantly alter the bone mineral profile, increasing the degree of tissue mineralization [74] and reducing its heterogeneity [76].

The use of BP for more than 3 years was shown to induce adverse changes (mainly an increase in nonenzymatic cross-links) within the bone's nonmineral organic matrix, specifically within the collagen fibers. This process leads the an increased formation and accumulation of AGEs, which induce alteration of collagen structure and disrupt its function [48, 51, 76]. BP-mediated reduction of bone remodelling may also result in a decreased removal of AGEs already accumulated, from the extracellular matrix, leading to premature skeletal ageing. Thus, the increased bone levels of AGEs have been associated with alterations in the mechanical bone properties, with a consequent decrease of bone quality [77–80].

Considering controversies from clinical studies [77], caution has been recommended for the use of BP as antiosteoporotic drugs in patients with diabetes mellitus. These limitations might be potentially related with AGEs that are increased in the extracellular matrix of bone tissue in patients with poorly compensated diabetes mellitus [76]. Therefore, BP might only partially affect AGE-mediated adverse effects on bone cells [72, 73, 81–83]. However, a direct effect of N-containing BP on AGEs has been also demonstrated [84]. On the other hand, Yamagishi and coworkers showed that Incadronate and Minodronate might indirectly revert deleterious effects of AGEs in human umbilical vein endothelial cells (HUVECs) [85, 86], via suppression of NADPH oxidase-derived intracellular reactive oxygen species (ROS) generation (usually required for AGEs/RAGE signaling in vascular cells).

Gangoiti and colleagues showed that other BP (such as alendronates, pamidronate, and zoledronate) might abrogate AGE-mediated effects on osteoblastic cells blocking ROS generation and Ca2+ influx through l-type voltage-sensitive channels [72]. More recently, the inhibitory activity of alendronate on AGE-mediated recruitment and differentiation of osteoclasts demonstrated a beneficial antiresorptive effects of BP when bone extracellular matrix accumulates excess AGEs. In cultured osteoblasts, coincubation of AGEs and alendronate reduces RANKL expression, suggesting a critical pathway for the inhibitory effects of these agents on the recruitment and differentiation of osteoclasts [73]. Similar results have been previously reported* in vitro* in both human and rat osteoblasts [37, 60, 70, 87–90] and in a clinical study enrolling women with postmenopausal osteoporosis [88].

Conversely, other studies demonstrated that the BP paradoxically increase AGE-mediated osteoclast resorptive action in the first 4 days of incubation [91], with a late inhibition after 8 days [91, 92]. Only high concentrations of alendronate (similar to the drug bone concentration) were shown to increase osteoblastic morphological and cytoskeletal alterations. On the other hand, lower doses of this drug did not affect cell morphology, but they are able to prevent the AGE-induced alterations in osteoblast morphology, apoptosis, and proliferation [73, 93].

Taken together, these studies suggest that severe consequences due to a long-term BP use might be related to AGE accumulation in bone via an increase in inflammation, ROS release, and bone turnover suppression. The damage appears to be offset by employing the lowest dosage of these drugs over a longer treatment time.

Therefore, especially in a young subject who starts BP and in other chronic inflammation conditions involving AGEs (such as diabetes and aging), we recommend using caution.

## 5. “Inflammaging” and Bone 

Human aging has been viewed as the declining function of the body systems and organs [94]. This process is the result of progressive damage of tissues and substances and the gradual loss of normal tissue and molecules. Considering inflammation, the characteristics of aging are (1) the progressive filling of the immunological system by activated lymphocytes, macrophages, and dendritic cells in response to chronic/continuous fine stress from pathological or physiological antigens/toxins accumulation and (2) the immunosenescence condition, characterized by a decreased ability of the immune system to respond to foreign antigens, as well as a decreased ability to maintain tolerance to self-antigens [95]. The term “inflammaging” is a coinage merging “inflammation” and “aging” proposed by the group of Professor Franceschi to describe the particular physical condition which provides a continuous mild antigenic challenge leading to a proinflammatory condition associated with the progressive stimulation/depletion of the immune system with aging [96]. For instance, increasing circulating levels of proinflammatory cytokines and C-reactive protein, which contribute to the maintenance of a low level of chronic inflammation, have been demonstrated also in healthy elderly individuals [9, 96–99]. This typical chronic inflammation has been described in age-related diseases, such as osteoporosis [11]. It is unclear whether the inflammatory state observed in many aging processes is responsible for the development of degenerative chronic diseases, or whether the chronic pathologies cause the inflammatory state observed in aging. Cumulative evidence indicated a tight, cause-effect link between oxidative stress, inflammaging, immunosenescence, and age-related diseases [100]. Oxidative stress has been indeed recognized to play a major role in determining and maintaining the low-grade inflammation typical of inflammaging [11]. The biochemical imbalance between the formation and clearance of oxidized proteins, lipids, and carbohydrates is also a main mechanism underlying inflammaging [101]. Oxidized proteins are often entangled in misfolded aggregates, which cannot be unfolded for proteasome degradation and form an “inclusion-like” body located in the cytosol. Among these oxidized molecular aggregates, AGEs have been demonstrated to accumulate within cells and participate to inflammation [101]. On the other hand, the enzymes in charge of free radical clearance in the cytosol (superoxide dismutase, catalase, and glutathione peroxidise) and those located in mitochondria (manganese superoxide dismutase) are decreased in aging cells, this contributing to the establishment of the chronic systemic inflammation in ageing [101].

In bone, chronic inflammation in aging has been described to increase pathogenetic factors in osteoporosis [6]. Experimental and clinical studies suggest that inflammation exerts a significant influence on bone turnover affecting the intrinsic balance of bone mineralization and resorption and supporting a link between the increased state of proinflammatory cytokines activity and the bone loss [10]. Clinical observations also reveal concomitance of systemic osteoporosis with events of systemic inflammation as well as colocalization of regional osteoporosis with areas of regional inflammation [6]. Indeed, several proinflammatory cytokines, such as IL-1, IL-6, TNF-*α*, and leukemia inhibitory factor (LIF) have been indicated as pro-osteoporotic mediators in both osteoblast and osteoclast regulation [9, 95, 102].

IL-1 is a potent stimulator of bone resorption [6, 103] and inhibitor of bone formation [104]; IL-6 and TNF-*α* were shown to promote osteoclast differentiation and activation and are involved in bone resorption. LIF, a paracrine/autocrine modulator of osteoblasts, stimulates bone resorption and increases the number of osteoclasts but also exerts anabolic effects on bone promoting osteoblast proliferation [105, 106]. These two different LIF-mediated actions seem to be due to its biphasic dose-response, which has already been demonstrated in vitro, with bone resorption dominating at high concentration and bone formation at lower doses [107]. On the other hand, although several studies confirmed the pathophysiological relevance of these cytokines, their role on osteoblasts in the development of bone loss remains to be elucidated. Taking into the account these evidences from basic research, the synthesis and accumulation of AGEs during “Inflammaging” might represent key events in the establishment and development of osteoporosis.

## 6. Conclusion

**Figure 1:**
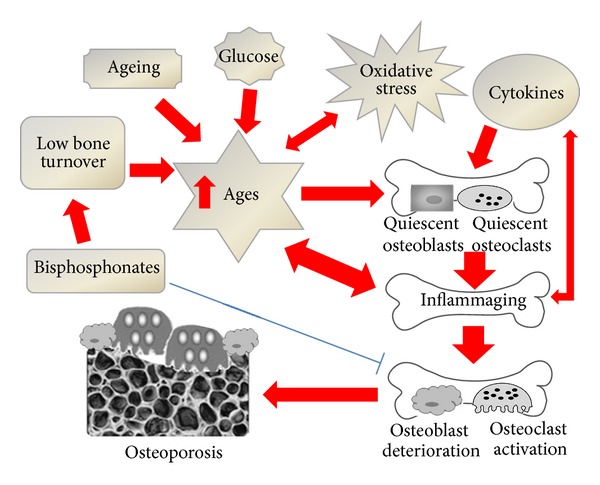
The vicious circle linking inflammation and aging results in the accumulation of AGEs within the osteoporotic bone. Oxidative stress, high glucose, aging processes, and low bone turnover conditions contribute to an increased formation and accumulation of AGEs in bone, where they trigger a low level of chronic inflammation defined as “Inflammaging.” Together with an increase of certain proinflammatory cytokines, AGEs induce both the activation of osteoclastogenesis and osteoblast dysfunction; these processes lead to an accelerated development of osteoporosis. The use of bisphosphonates might have a dual effect: it inhibits osteoclastogenesis by improving the bone resorption, but it slows the bone turnover, increasing the accumulation of AGEs and potential long term adverse effects.

Aging is characterized by a decline of anatomical integrity and function across multiple organ system and a reduced ability to respond to stress. The multisystem decline is associated with increasing pathology, disease, and progressively higher risk of death. Among the age-related chronic degenerative diseases, osteoporosis represents a major challenge to health care services, particularly with increases in the elderly population worldwide. Indeed, in addition to fractures, osteoporosis carries a considerably risk of disability due to serious medical complication so, with the aging of population, its prevalence is expected to increase in developed countries and consequently its already heavy medical, social, and financial burdens. Osteoporosis is also associated with a low level of chronic inflammation. This inflammatory state associated with immunosenescence is defined as “inflammaging” and is characterized by generation of oxidative stress mediators (including AGEs) and proinflammatory cytokines. AGEs accumulate in the bone tissue, due to its low turnover and the content of long life-time proteins, such as collagen. The modification of these proteins by AGEs is clearly implicated in the development of osteoporosis (Figure 1). AGEs might also directly increase osteoporosis via their binding to the specific receptor RAGE expressed on both bone and inflammatory cells. Thus, osteoblast and osteoclast differentiation, maturation, and function might be directly influenced by AGEs (Figure 1). In particular, the compounds were shown to lower the capacity of osteoblasts to form normal bone and increase osteoclastogenic potential, thus favouring osteoporosis.

Although scientific evidence requires additional studies, the use of potent antiosteoporotic drugs (such as BP) has to be carefully evaluated in clinical conditions characterized by AGE accumulation. In fact, the low bone turnover due to osteoclastogenesis suppression might paradoxically favour the establishment of oxidative damage and chronic inflammation, potentially worsening (at least in the first days of treatment) the bone tissue stability.

We believe that a better understanding of molecular mechanisms by which AGEs trigger impairment in bone function and structure might allow the identification of more selective treatments to prevent and treat adverse “Inflammaging” in osteoporosis.
